# Feasibility of High-dose Iodine-131-metaiodobenzylguanidine Therapy for High-risk Neuroblastoma Preceding Myeloablative Chemotherapy and Hematopoietic Stem Cell Transplantation: a Study Protocol

**DOI:** 10.22038/aojnmb.2018.29845.1203

**Published:** 2018

**Authors:** Raita Araki, Ryosei Nishimura, Anri Inaki, Hiroshi Wakabayashi, Yasuhito Imai, Yoshikazu Kuribayashi, Kenichi Yoshimura, Toshinori Murayama, Seigo Kinuya

**Affiliations:** 1 Department of Pediatrics, School of Medicine, Institute of Medical, Pharmaceutical and Health Sciences, Kanazawa University, Japan; 2 Department of Nuclear Medicine, School of Medicine, Institute of Medical, Pharmaceutical and Health Sciences, Kanazawa University, Japan; 3 Innovative Clinical Research Center, Kanazawa University, Japan

**Keywords:** ^131^I-MIBG, Hematopoietic stem cell transplantation, Neuroblastoma, Prospective study protocol

## Abstract

**Objective(s):**

High-risk neuroblastoma is a childhood cancer with poor prognosis despite modern multimodality therapy. Internal radiotherapy using ^131^I-metaiodobenzylguanidine (MIBG) is effective for treating the disease even if it is resistant to chemotherapy. The aim of this study is to evaluate the safety and efficacy of ^131^I-MIBG radiotherapy combined with myeloablative high-dose chemotherapy and hematopoietic stem cell transplantation.

**Methods:**

Patients with high-risk neuroblastoma will be enrolled in this study. A total of 8 patients will be registered. Patients will receive 666 MBq/kg of ^131^I-MIBG and after safety evaluation will undergo high-dose chemotherapy and hematopoietic stem cell transplantation. Autologous and allogeneic stem cell sources will be accepted. After engraftment or 28 days after hematopoietic stem cell transplantation, the safety and response will be evaluated.

**Conclusion:**

This is the first prospective study of ^131^I-MIBG with high-dose chemotherapy and hematopoietic stem cell transplantation in Japan. The results will be the basis of a future nationwide clinical trial.

## Introduction

Neuroblastoma is the most common extra-cranial solid tumor in childhood and has a broad spectrum of clinical behavior (1). A median age at diagnosis is about 19 months, and 98% of neuroblastoma patients are diagnosed by 10 years of age (2). Patients are stratified according to the risk of relapse, which is dependent on age at diagnosis, clinical stage, and biological features including *MYCN* oncogene amplification. Current treatment for high-risk disease, which has the worst prognosis (3), consists of induction chemotherapy and surgery, consolidation therapy with high-dose chemotherapy (HDC) with autologous hematopoietic stem-cell transplantation (autologous HSCT) and irradiation, and post-consolidation therapy. Despite modern multimodality therapy, event-free survival for high-risk disease is less than 50% (3, 4). A median time to disease recurrence is reported to be 18.3 months since initial diagnosis, and the survival rate after relapse is particularly poor (~10%) (5, 6). Clearly, novel and more effective therapeutic strategies are required for high-risk disease.

Most patients (~90%) with neuroblastoma have tumors that accumulate metaiodobenzylguanidine (MIBG), an analog of norepinephrine, via the norepinephrine transporter. Hence, radiolabeled MIBG is used for imaging studies and targeted therapy (7). Dose escalation studies of ^131^I-MIBG single-agent therapy revealed that a high-dose was required for response, with 666 MBq/kg considered to be the clinically maximum dose (8, 9). Although non-hematological toxicities were rare and mild, severe hematological toxicities were frequent and required hematological stem-cell rescue in 36% of cases (10). Subsequently, combined therapy with HDC and autologous HSCT was investigated using 444 MBq/kg of ^131^I-MIBG combined with carboplatin, etoposide, and melphalan. This approach demonstrated that combined therapy was feasible and effective in primary refractory neuroblastoma (11). However, a prospective study combining 666 MBq/kg of ^131^I-MIBG with HDC is scarce so far. Furthermore, there is great concern about toxicities associated with ^131^I-MIBG and HDC treatment of relapse patients. Therefore, prospective studies are needed to assess the feasibility of the clinically maximum dose of ^131^I-MIBG combined with HDC not only for consolidation therapy of newly diagnosed high-risk patients, but also for relapse patients.

## Methods

***Study outline***

**Figure 1:**
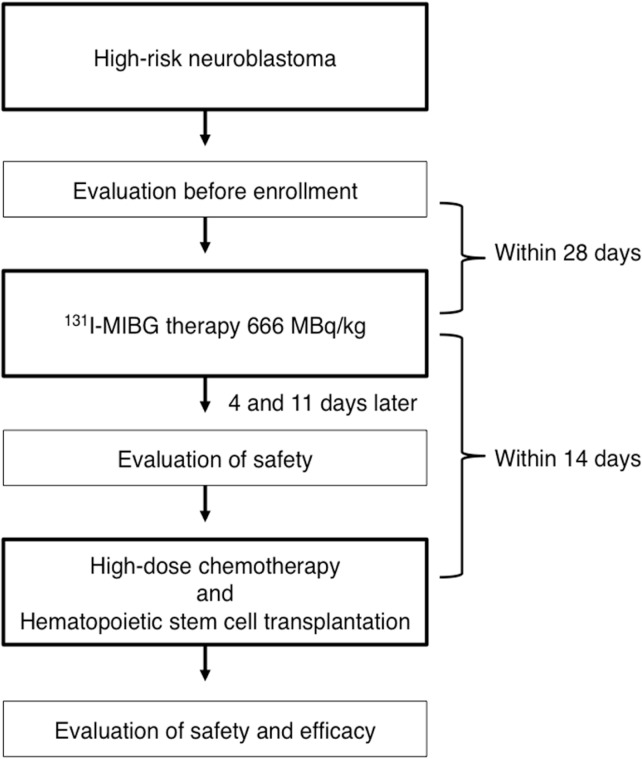
Study outline

The study participants will be newly diagnosed or relapsed high-risk neuroblastoma patients. All participants will receive 666 MBq/kg of ^131^I-MIBG. After a radiation isolation period, adverse effects and organ function will be evaluated. The participants who meet the safety criteria will undergo HDC and HSCT. Engraftment, safety, and response to therapy will be assessed (Figure 1).

***Purpose***

The objective of this study is to evaluate the safety and efficacy of ^131^I-MIBG radiotherapy when it is administered prior to HDC and HSCT for patients with high-risk neuroblastoma.

***Study design***

The study is an open-label, single institutional single arm clinical trial.

***Ethical considerations and registration***

This study will be conducted in accordance with the Declaration of Helsinki and the International Committee for Harmonization of Good Clinical Practice guidelines. The Institutional Review Board of Kanazawa University Hospital approved the study. Written informed consent will be obtained from all participants or their guardian before registration. This study was registered with the UMIN clinical Trial Registry (UMIN000025045).

***Endpoint***

The primary endpoint will be dose-limiting toxicity (DLT). DLT is defined as any adverse events associated with ^131^I-MIBG that comprise a major obstacle to subsequent HDC including exacerbation of Eastern Cooperative Oncology Group (ECOG) performance status scale ≥2, oxygen desaturation <94%, toxicities grade ≥3, serum creatinine level above the reference value according to age, creatinine clearance <70 ml/min/1.73 m^2^, active infection, and any other adverse events that comprise a major obstacle to HDC and HSCT. Toxicities will be graded according to the National Cancer Institute Common Terminology Criteria for Adverse Events (CTCAE) version 4.0. DLT will be evaluated 4 and 11 days after MIBG administration.

The secondary endpoints will be incidence and type of adverse events, hematopoietic stem cell engraftment rate, response rate according to the Response Evaluation Criteria in Solid Tumors (RECIST) and ^123^I-MIBG scintigraphy, overall survival, and progression free survival.

***Eligibility criteria***

***Inclusion criteria***

Prior to enrollment, patients must meet all of the following inclusion criteria (this study has no age limit, but has body weight limit as mentioned in exclusion criteria): definitive diagnosis of neuroblastoma; newly diagnosed or relapsed patients with high-risk neuroblastoma according to Children’s Oncology Group (COG) or International Neuroblastoma Risk Group (INRG) classification; one or more lesions accumulating ^123^I-MIBG at initial presentation or relapse; cryopreserved autologous peripheral blood stem cells, cord blood, or other stem cell sources must be available; neutrophil count ≥500/µl, platelet count ≥20×10^9^/l, hemoglobin concentration ≥7.0 g/dl, serum creatinine level below the reference value according to age, creatinine clearance ≥70 ml/min/1.73m^2^, alanine aminotransferase (ALT) ≤5× upper limit of normal for age, aspartate aminotransferase (AST) ≤5× upper limit of normal for age, total bilirubin ≤3× upper limit of normal for age, New York Heart Association (NYHA) classification class I or below, oxygen saturation ≥94%; ECOG performance status 0 or 1. In addition, patients must be able to cope with radiation safety isolation and provide written informed consent by patients or guardian.

***Exclusion criteria***

Patients who meet any of the following criteria will be excluded: active double cancer; diffuse bone marrow involvement on ^123^I-MIBG scintigraphy; progressive disease; hepatitis B virus (HBV) (or carrier), hepatitis C virus (HCV), human immunodeficiency virus (HIV), or other active infection; history of fatal arrhythmia or asystole; concurrent poorly controlled symptomatic arrhythmia, thyroid dysfunction, respiratory disorder, pleural effusion, or ascites; concurrent coronary artery disease, usage of amiodarone, severe cardiac valvulopathy, aortic disease, or bleeding tendency; woman during pregnancy or lactation, within 28 days postpartum, or desiring pregnancy within 1 year; concurrent poorly controlled psychiatric disorder; allergy to potassium iodide; difficulty coping with radiation safety isolation; concurrent palliative external radiotherapy to painful lesions; past treatment by the same regimen as this study; unable to receive at least of 444 MBq/kg of MIBG due to exceeding the upper limit of radioisotope use at the center (as the upper limit at our center is 24,000 MBq, patients over 54 kg are excluded); patients who may not be able to comply with the requirements of the study, including drug adherence, instruction by medical staffs, and cooperation for various examinations.

***Patient registration***

The investigators will send a patient registration form to the independent data center at the academic research organization at Kanazawa University Hospital. Patient registration began in July 2017 and will be complete in April 2019.

***Treatment***

^131^I-MIBG therapy was planned according to the Japanese draft guidelines regarding appropriate use of ^131^I-MIBG radiotherapy for neuroendocrine tumors by the Guideline Drafting Committee for Radiotherapy with ^131^I-MIBG, Committee for Nuclear Oncology and Immunology, the Japanese Society of Nuclear Medicine (JSNM) (12) and the procedure guidelines for ^131^I-MIBG therapy from the European Association of Nuclear Medicine (EANM) (13). In a radiation isolation room, patients receive 666 MBq/kg of ^131^I-MIBG intravenously over an hour at day 0. If the dosage exceeds the upper limit of radioisotope use (24,000 MBq), a decrease to 444 MBq/kg is permitted. Subsequently, HDC and HSCT are initiated within 2 weeks after ^131^I-MIBG administrations. The recommended regimens are CEM (carboplatin, etoposide, and melphalan) for autologous HSCT, or BuMel (busulfan and melphalan) for autologous or allogeneic HSCT. Other regimens including reduced intensity stem cell transplantation are permitted depending on the patient’s condition and a selected stem cell source. Both autologous and allogeneic hematopoietic stem cells are approved as a stem cell source.

***Prescribed, recommended or acceptable supportive treatments***

Potassium iodide will be administered orally to prevent the thyroidal uptake of free iodide. Patients will take potassium iodide 300 mg/day from at least 24 hours before ^131^I-MIBG infusion, and it will be continued until 7 days after the infusion. 5-HT3 receptor antagonist is recommended during ^131^I-MIBG therapy, and given according to each drug information. Supportive treatment during HDC and HSCT will be performed according to institutional guidelines. Recommended prophylaxis for graft-versus-host disease is a combination of tacrolimus and short-term methotrexate. Tacrolimus will be intravenously administered with 0.02-0.03 mg/kg/day from the day before HSCT. Methotrexate will be intravenously administered with 15 mg/m^2^ on day 1, and with 10 mg/m^2^ on day 3, 6, and 11 after HSCT. The modification of prophylaxis for graft-versus-host disease will be accepted depending on the preconditioning regimen and the selected stem cell source.

***Schedule of evaluation***

The study period is from the date of enrollment to the date of evaluation after hematopoietic stem cell engraftment. Safety is evaluated at enrollment, before ^131^I-MIBG therapy, during the radiation isolation period, at day 4 and 11, before HDC, after engraftment of hematopoietic stem cells or 28 days after HSCT. Tumor markers and imaging studies after engraftment or 28 days after HSCT are compared with baseline to evaluate the efficacy of this study.

 The independent committee for evaluation of safety and efficacy will be convened if unexpected severe adverse reactions occur. All severe adverse events including death for any reason, unexpected admission, and unexpected prolonged admission will be immediately reported to the Ministry of Health, Labour and Welfare.

***Sample size***

The sample size is set at 8 patients (with 6 treated patients needed). Considering that it is possible to obtain the same level of precision as that required in a Phase I trial of general anticancer drugs, the number of treated patients was set to 6. 98% of neuroblastoma patients are diagnosed by 10 years of age, and the average body weight of 10-year-old Japanese children is 33.2 kg for boys and 32.8 kg for girls. Therefore we can expect at least 6 participants with 666 MBq/kg. 

***Statistical analysis***

The incidence of DLT will be evaluated in the primary analysis. If the DLT is not observed in two or more patients, high-dose ^131^I-MIBG therapy is concluded to be safe and feasible for the patients with high-risk neuroblastoma preceding myeloablative chemotherapy and HSCT. The confidence interval of DLT incidence is estimated based on a binomial distribution. The data from the participants with reduced ^131^I-MIBG will be analyzed separately from those with 666 MBq/kg.

## Discussion

This is the first prospective study to evaluate the safety and efficacy of ^131^I-MIBG therapy combined with HDC and HSCT for high-risk neuroblastoma in Japan. The dosage of ^131^I-MIBG employed in this study is 666 MBq/kg, which is the maximum dose for single-agent therapy (8, 9). The benefit of the ^131^I-MIBG and HDC is that planned HSCT rescues myeloablation not only from HDC but also from ^131^I-MIBG radiotherapy. On the other hand, any adverse events of ^131^I-MIBG that comprise a major obstacle to subsequent HDC are a serious drawback of this combination therapy. This occurrence will be evaluated as a primary endpoint.

Neuroblastoma is highly sensitive to radiation therapy. Indeed, external radiation therapy was reported to control local bone metastasis in 74% of lesions (14). However, lesions that are widely distributed or adjacent to critical organs are not suitable for external radiation. Total body irradiation (TBI) has also been employed previously as an HDC regimen for disease control. However, due to its toxicity the TBI regimen has now been superseded by the non-TBI regimen.(15, 16, 17). To compensate for the disadvantage of external irradiation, internal irradiation with ^131^I-MIBG is considered most suitable for refractory high risk neuroblastoma. Several reports demonstrated that ^131^I-MIBG is effective in diseases resistant to chemotherapy (18). In addition, with the exception of thyroid dysfunction, non-hematologic toxicity of ^131^I-MIBG is reported to be rare (19, 20). Therefore, the ^131^I-MIBG containing regimen is attractive in view of its efficacy and minimal toxicity.

Another important aspect of this study is that it includes relapsed patients and accepts allogeneic stem cell sources. Most relapsed patients have received previous intensive chemotherapy and HDC as their initial treatments. Given the organ damage from their previous treatments, second HDC and HSCT are challenging. In this study, strict criteria about organ function at registration ensure the safety of relapsed patients. Autologous HSCT is standard for high-risk neuroblastoma. However, promising results using allogeneic HSCT to promote graft-versus-tumor responses have stimulated the launch of new clinical trials (21). To date, only a small case series of combination therapy with ^131^I-MIBG and allogeneic HSCT has been reported (22). These workers used grafts from HLA-haploidentical donors only, and concluded their approach was limited due to unacceptable graft-versus-host disease. Our study approves HLA-matched related or unrelated donor and cord blood, which may be less prone to graft-versus-host disease compared with HLA-haploidentical donors. Therefore transplant-related morbidity and mortality is expected to be acceptable. Data on engraftment, graft-versus-host disease, graft-versus-tumor effect, and other transplant-related complications will be obtained in this study. If the protocol is successful, children with high-risk neuroblastoma will have more treatment choices.

## Conclusion

This is the first prospective study of ^131^I-MIBG with HDC and HSCT in Japan. The results will be the basis of a future nationwide clinical trial.
